# Network Pharmacology‐Based Identification of Potential Targets and Mechanisms of Isoginkgetin in Gastric Cancer

**DOI:** 10.1155/bmri/8863892

**Published:** 2026-06-16

**Authors:** Linen Li, Huiling Zhu, Kun Cao, Hao Chen

**Affiliations:** ^1^ Department of Gastroenterology, Shangrao People′s Hospital, Shangrao, Jiangxi, China; ^2^ Department of Gastroenterology, The First Affiliated Hospital of Nanchang University, Nanchang, Jiangxi, China, ncu.edu.cn; ^3^ Department of Gastroenterology, The Central Hospital of Jingmen, Jingmen, Hubei, China

**Keywords:** cell cycle, gastric cancer, ISO, network pharmacology

## Abstract

**Background:**

Isoginkgetin (ISO) is a natural flavonoid with potential anticancer effects. However, the anticancer mechanisms of ISO in gastric cancer remain insufficiently explored.

**Methods:**

Network pharmacology, TCGA data analysis, and molecular docking were employed to identify potential ISO targets and their involvement in gastric cancer. Differentially expressed genes (DEGs) in gastric cancer were compared with ISO targets to find overlapping genes. Functional enrichment analysis was conducted to determine key biological pathways. Cytoscape‐MCODE was used to identify hub genes, and molecular docking assessed the binding stability between ISO and these hub genes.

**Results:**

A total of 107 potential ISO targets were identified, of which 66 overlapped with gastric cancer DEGs. Functional enrichment analysis indicated involvement in cell cycle regulation, cellular senescence, and the PI3K–Akt signaling pathway. Ten hub genes (GSK3B, TERT, CCNT1, CCNB3, CCNB2, CREB1, CCNA2, CCNB1, CDK1, and ESR1) were identified using Cytoscape‐MCODE. Most genes were highly expressed in tumor tissues and positively correlated, with some significantly associated with patient survival. Molecular docking demonstrated stable binding between ISO and the hub genes.

**Conclusion:**

In conclusion, this study systematically explored the molecular targets and potential mechanisms of ISO in gastric cancer, providing a preliminary theoretical basis for further research.

## 1. Introduction

Gastric cancer (GC) remains one of the most common and lethal malignancies worldwide, with high incidence and poor prognosis [1]. Despite advances in surgical techniques, chemotherapy, and targeted therapies, the overall survival rate of GC patients remains unsatisfactory [2–4], highlighting the urgent need for novel therapeutic agents and molecular targets.

Natural compounds have been shown to possess significant anticancer properties. Research has clarified both their efficacy and molecular mechanisms, forming a foundation for their application in cancer treatment [5]. Isoginkgetin (ISO), a naturally occurring flavonoid, has been reported to possess multiple pharmacological effects, including anti‐inflammatory, antioxidant, and anticancer activities. In terms of anticancer effects, in one study, ISO was found to inhibit the proliferation and migration of U87MG glioblastoma cells by activating apoptosis and autophagy [6]. In another study, ISO, as a potential CDK6 inhibitor, suppressed SLC2A1/GLUT1 enhancer activity, thereby inducing AMPK‐ULK1–mediated cytotoxic autophagy and exerting inhibitory effects on hepatocellular carcinoma [7]. However, the molecular mechanisms underlying ISO′s anticancer effects, particularly in GC, remain largely unexplored.

Network pharmacology has emerged as a powerful strategy to systematically investigate the interactions between bioactive compounds and disease‐related targets, providing insights into the potential mechanisms of action at the molecular and pathway levels [8]. Combined with bioinformatics analyses of high‐throughput datasets, such as The Cancer Genome Atlas (TCGA), and molecular docking approaches, this strategy enables the identification of key targets and signaling pathways that may mediate therapeutic effects. In this study, we applied a comprehensive approach integrating network pharmacology, differential gene expression analysis, protein–protein interaction (PPI) network construction, functional enrichment analysis, and molecular docking to elucidate the potential targets and mechanisms of ISO in GC. We further investigated the expression patterns, interrelationships, and prognostic significance of hub genes in tumor and normal tissues, aiming to provide a theoretical basis for the development of ISO as a potential therapeutic agent for GC.

## 2. Materials and Methods

### 2.1. Retrieval of ISO Structure and Target Prediction

The 2D structure and SMILES of ISO were obtained from the PubChem database (https://pubchem.ncbi.nlm.nih.gov/). Potential targets of ISO were predicted using SwissTargetPrediction (http://www.swisstargetprediction.ch/) and the Similarity Ensemble Approach (SEA, http://sea.bkslab.org/). After removing duplicates, the candidate target list was used for subsequent analyses.

### 2.2. Differential Expression Analysis in GC

RNA‐seq data of GC and normal gastric tissues were obtained from TCGA‐STAD (https://portal.gdc.cancer.gov/). Expression data were normalized, and differential expression analysis was performed using the DEseq2 in R [9]. DEGs were identified using |log2 *f* *o* *l* *d* *c* *h* *a* *n* *g* *e*| and adjusted *p* value (FDR) thresholds, and visualized by volcano plots and heatmaps.

### 2.3. Intersection of ISO Targets and DEGs

Predicted ISO targets were intersected with DEGs to identify overlapping genes, which were visualized by Venn diagrams using bioinformatics tools (https://www.bioinformatics.com.cn/). These overlapping genes were used for PPI network construction and functional analyses.

### 2.4. PPI Network Construction and Hub Gene Identification

Overlapping genes were input into the STRING database (https://string-db.org/) [10] with *Homo sapiens* as the species. The resulting PPI network was visualized in Cytoscape 3.9.0. Hub genes were identified using the MCODE [11] plugin based on topological parameters such as degree.

### 2.5. Gene Ontology (GO) and KEGG Enrichment Analyses

R package “clusterProfiler” [12] was used for GO and KEGG pathway enrichment of overlapping and hub genes. GO analysis included biological process (BP), cellular component (CC), and molecular function (MF). *p* < 0.05 was considered statistically significant, and results were visualized.

### 2.6. Hub Gene Expression and Correlation Analysis

TCGA‐STAD data were used to compare expression levels of hub genes in tumor versus normal tissues using boxplots. Pairwise correlations among hub genes were calculated and visualized as a correlation heatmap.

### 2.7. Clinical Feature Analysis of Hub Gene Expression

UALCAN (https://ualcan.path.uab.edu/) [13] was used to analyze hub gene expression across different GC clinical stages, tumor grades, and lymph node metastasis statuses.

### 2.8. Survival Analysis

Kaplan–Meier Plotter (https://kmplot.com/analysis/) [14, 15] was used to evaluate the association between hub gene expression and overall survival in GC. Patients were stratified into high‐ and low‐expression groups by median expression. Log‐rank tests were applied, with *p* < 0.05 considered significant.

### 2.9. Molecular Docking

ISO was used as the ligand, and 10 hub gene–encoded proteins were used as receptors. Protein structures were obtained from the Protein Data Bank (PDB) (https://www.rcsb.org/) [16] and preprocessed by removing water molecules and ligands, adding hydrogens, and assigning charges. Docking simulations were performed, and binding energies were calculated. Molecular docking experiments were performed using AutoDockTools [17] to investigate the binding interactions between ISO and its target proteins. The docking results were visualized using PyMOL [18] to illustrate the molecular interactions involved. Docking conformations and hydrogen bond interactions were visualized to assess binding affinity.

### 2.10. Molecular Dynamics (MD) Simulations

The CDK1/cyclin B–ISO and GSK3B–ISO complex systems were analyzed using a consistent workflow: The protein was modeled using the ff14SB force field, whereas the ligand was parameterized with the GAFF2 force field. The system was solvated in an explicit TIP3P water model. Appropriate Na^+^/Cl^−^ ions were subsequently added to neutralize the system and mimic physiological ionic strength. MD simulations were carried out following a standard protocol, including energy minimization, NVT equilibration, NPT equilibration, and a 100‐ns production run. Based on the production trajectories, structural stability and conformational dynamics of the system were systematically analyzed, including backbone root mean square deviation (RMSD), C*α* root mean square fluctuation (RMSF), radius of gyration (Rg), protein–ligand hydrogen bonds, solvent‐accessible surface area (SASA), and free energy landscape (FEL).

## 3. Result

### 3.1. Identification and Characterization of ISO Targets and Differentially Expressed Genes

**Figure 1:**
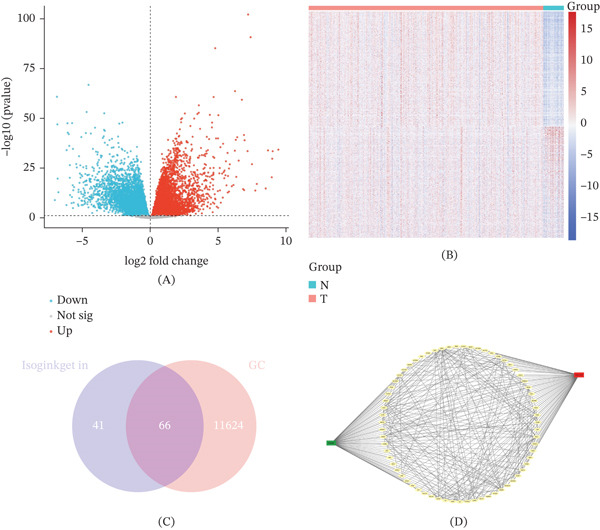
Identification of ISO targets and differentially expressed genes (DEGs) in gastric cancer. (A) Volcano plot showing DEGs between gastric cancer tissues and normal tissues based on TCGA‐STAD data. Red dots represent upregulated genes, blue dots represent downregulated genes, and gray dots represent nonsignificant genes. (B) Heatmap of DEGs. (C) Venn diagram showing the overlap between predicted ISO targets and DEGs, resulting in 66 common genes. (D) Protein–protein interaction (PPI) network of overlapping genes constructed using STRING and visualized in Cytoscape 3.9.0.

The two‐dimensional structure and SMILES notation of ISO were retrieved from the PubChem database and subsequently analyzed using the SwissTargetPrediction and SEA databases to identify potential molecular targets, resulting in 107 candidate targets (Table S1). Based on TCGA‐STAD data, differential expression analysis comparing normal tissues with GC samples revealed a total of 11,690 differentially expressed genes, which were illustrated by volcano plots and heatmaps (Figure 1A,B). By intersecting the predicted ISO targets with these differentially expressed genes, 66 common genes were identified (Figure 1C) (Table S2). These overlapping genes were then used to establish a PPI network based on the STRING database, and the network was visualized using Cytoscape 3.9.0 (Figure 1D).

### 3.2. Functional Enrichment of Overlapping Genes

**Figure 2:**
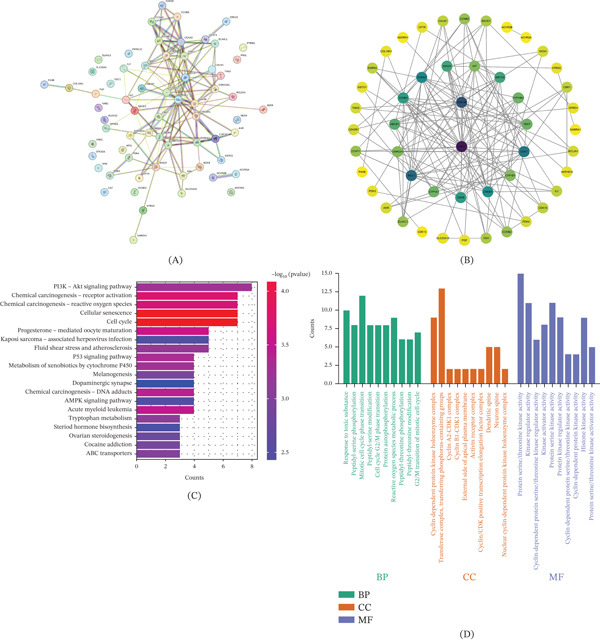
Functional enrichment analysis of overlapping genes. (A–B) Regulatory network of overlapping genes, with node colors representing interaction strength (darker color indicates stronger relevance). (C) KEGG pathway enrichment of the overlapping genes. (D) GO enrichment analysis showing significant biological processes, cellular components, and molecular functions associated with the overlapping genes.

Figure 2A,B depicts the regulatory network of the overlapping genes, emphasizing the PPI between them. This network offers valuable information regarding the functional associations and possible pathways related to these genes. In Figure 2B, variations in color intensity represent the relative strength of interactions, with deeper colors indicating stronger interaction relevance. GO and KEGG enrichment analyses of the 66 overlapping genes were conducted using the “clusterProfiler” package. The KEGG results demonstrated that these genes were markedly enriched in multiple pathways, including cellular senescence, cell cycle, chemical carcinogenesis–receptor activation, chemical carcinogenesis–reactive oxygen species, and the PI3K–Akt signaling pathway, among others (Figure 2C). According to GO enrichment analysis, the BP category showed significant enrichment in responses to toxic substances, peptidyl‐serine phosphorylation and modification, mitotic cell cycle phase transition, G2/M phase transition of the cell cycle, protein autophosphorylation, reactive oxygen species metabolic processes, peptidyl‐threonine phosphorylation and modification, and the G2/M transition of the mitotic cell cycle. In terms of CCs, these genes were mainly associated with serine/threonine protein kinase complexes, protein kinase complexes, cyclin‐dependent protein kinase holoenzyme complexes, transferase complexes involved in phosphorus‐containing group transfer, cyclin A2–CDK1 complexes, cyclin B1–CDK1 complexes, the external side of the apical plasma membrane, activin receptor complexes, cyclin/CDK positive transcription elongation factor complexes, and dendritic spines. Regarding MFs, significant enrichment was observed in protein serine/threonine kinase activity, kinase regulator and activator activities, cyclin‐dependent protein serine/threonine kinase regulatory activity, protein serine kinase activity, protein kinase regulatory activity, cyclin‐dependent protein serine/threonine kinase activity, cyclin‐dependent protein kinase activity, histone kinase activity, and protein serine/threonine kinase activator activity (Figure 2D). Collectively, these results suggest that ISO may influence the progression of GC by regulating cell cycle processes.

### 3.3. Identification of Hub Genes and Functional Enrichment

**Figure 3:**
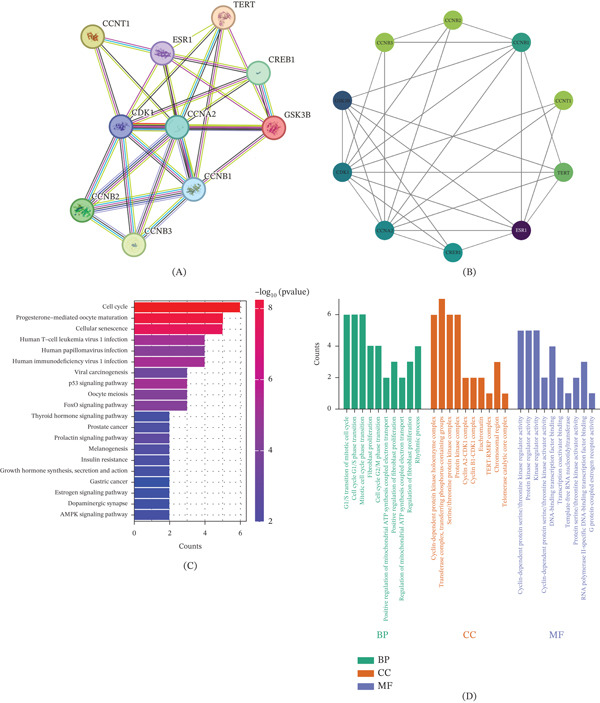
Identification and functional enrichment of hub genes. (A–B) PPI network of 66 overlapping genes analyzed with Cytoscape‐MCODE to identify the top 10 hub genes. Node color indicates node degree. (C) KEGG enrichment of the hub genes. (D) GO enrichment of hub genes, including biological processes, cellular components, and molecular functions.

**Figure 4:**
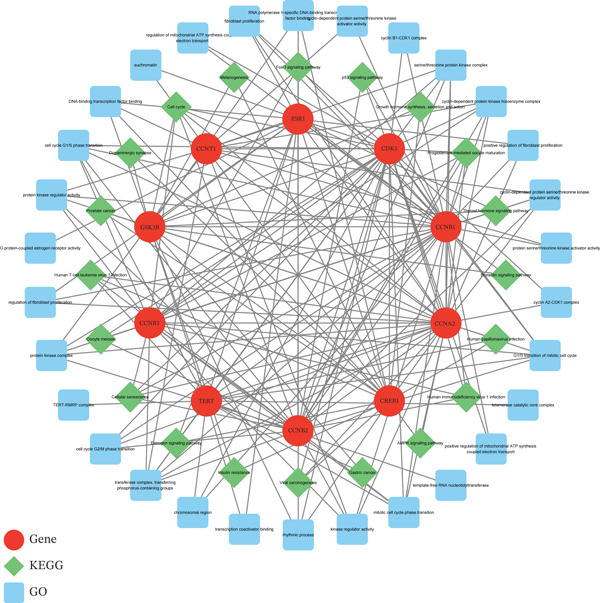
Target–pathway network of ISO in gastric cancer. The network illustrates the relationship between ISO, its core targets, and the KEGG and GO‐enriched pathways. Nodes represent genes or pathways, and edges indicate regulatory associations.

Using Cytoscape combined with the MCODE plugin, the top 10 hub genes were identified: GSK3B (glycogen synthase kinase 3 beta), TERT (telomerase reverse transcriptase), CCNT1 (cyclin T1), CCNB3, CCNB2, CREB1 (cAMP response element‐binding protein 1), CCNA2 (cyclin A2), CCNB1, CDK1 (cyclin‐dependent kinase 1), and ESR1 (estrogen receptor alpha) (Figure 3A,B; Table 1). These genes were ranked based on their node degree scores, with the node colors reflecting the degree values. GO and KEGG enrichment analyses were conducted on these 10 hub genes. KEGG results showed that they were significantly enriched in pathways including the cell cycle, progesterone‐mediated oocyte maturation, cellular senescence, human T‐cell leukemia virus 1 infection, and human papillomavirus infection, among others (Figure 3C). GO analysis results showed that these genes were significantly enriched in BPs including the G1/S transition of the mitotic cell cycle, cell cycle G1/S phase transition, mitotic cell cycle phase transition, fibroblast proliferation, cell cycle G2/M phase transition, positive regulation of mitochondrial ATP synthesis coupled electron transport, positive regulation of fibroblast proliferation, regulation of mitochondrial ATP synthesis coupled electron transport, regulation of fibroblast proliferation, and rhythmic processes, among others. In terms of CCs, the genes were mainly enriched in cyclin‐dependent protein kinase holoenzyme complexes, transferase complexes transferring phosphorus‐containing groups, serine/threonine protein kinase complexes, protein kinase complexes, cyclin A2–CDK1 complexes, cyclin B1–CDK1 complexes, euchromatin, TERT–RMRP complexes, chromosomal regions, and the telomerase catalytic core complex, among others. For MFs, significant enrichment was observed in cyclin‐dependent protein serine/threonine kinase regulator activity, protein kinase regulator activity, kinase regulator activity, cyclin‐dependent protein serine/threonine kinase activator activity, DNA‐binding transcription factor binding, transcription coactivator binding, template‐free RNA nucleotidyltransferase activity, protein serine/threonine kinase activator activity, RNA polymerase II‐specific DNA‐binding transcription factor binding, and G protein‐coupled estrogen receptor activity, among others (Figure 3D). In addition, based on the core targets corresponding to ISO and the predicted KEGG and GO enrichment results, a target–pathway relationship network diagram was constructed (Figure 4).

**Table 1:** HUB genes identified by MCODE.

Gene	Score
GSK3B	4.46
TERT	4.76
CCNT1	5
CCNB3	5
CCNB2	5
CREB1	5
CCNA2	5.57
CCNB1	5.57
CDK1	5.57
ESR1	5.57

### 3.4. Comparative Analysis of the Expression Patterns and Interrelationships of 10 Hub Targets in Normal and Tumor Tissues

**Figure 5:**
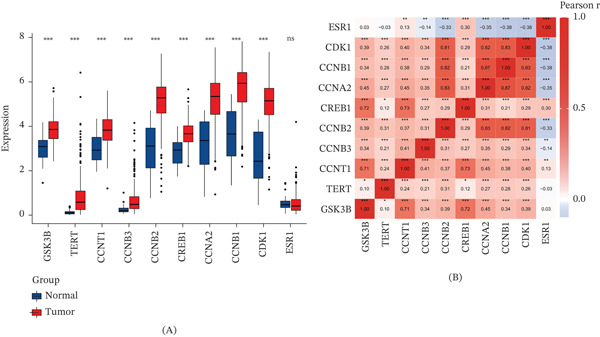
Expression patterns and correlations of hub genes in GC and normal tissues. (A) Boxplots showing expression levels of hub genes in gastric cancer and normal tissues. (B) Correlation heatmap demonstrating positive correlations among all 10 hub genes.

Based on TCGA‐STAD data, we analyzed the expression levels and correlations of 10 hub targets in GC and normal tissues. The analysis revealed that GSK3B, TERT, CCNT1, CCNB3, CCNB2, CREB1, CCNA2, CCNB1, and CDK1 were significantly upregulated in tumor tissues compared with normal tissues (Figure 5A). Moreover, all these genes exhibited positive correlations with each other (Figure 5B). Using publicly available databases such as UALCAN, the gene expression levels of the top 10 hub targets in GC patients were analyzed across advanced cancer stages, higher tumor grades, and metastatic lymph node statuses (Figure 6). These findings reveal distinct expression patterns of hub targets across different stages of GC progression.

Figure 6Expression of hub genes across different clinical stages of GC. Expression analysis using UALCAN database shows hub gene expression in relation to tumor stage, grade, and lymph node metastasis.

**Figure:**
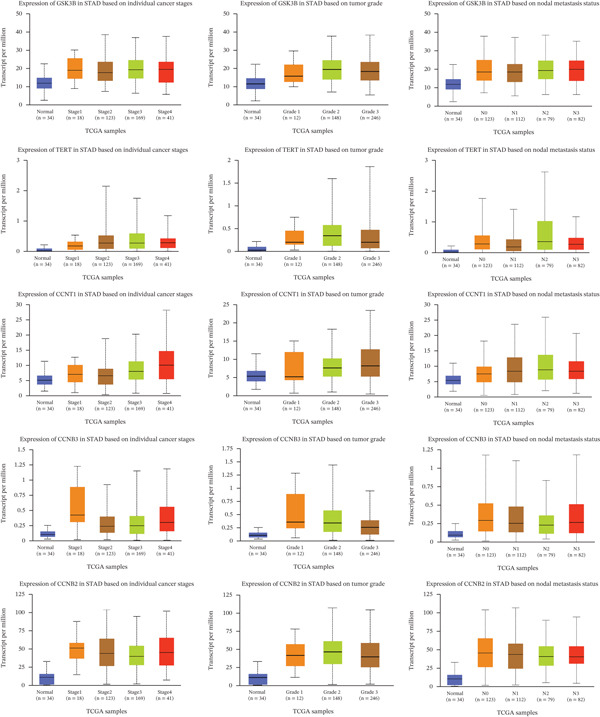
(A)

**Figure:**
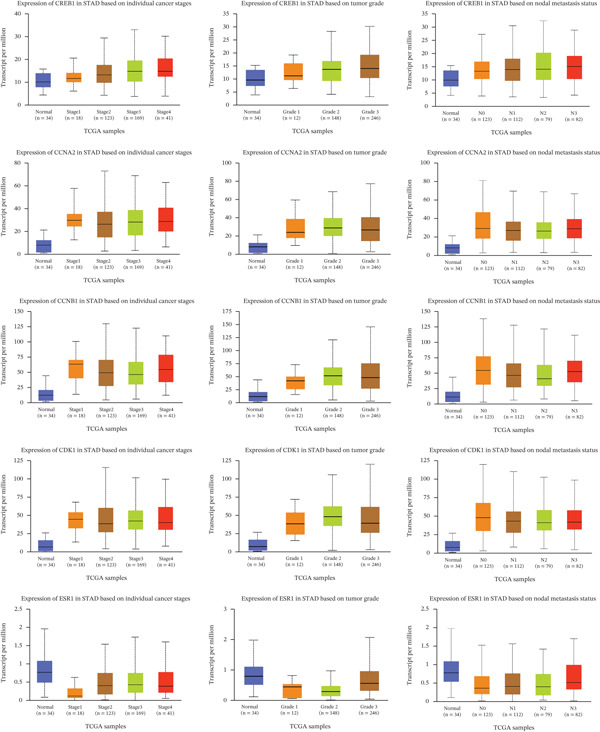
(B)

### 3.5. Survival Analysis of Hub Genes in GC and Normal Tissues

**Figure 7:**
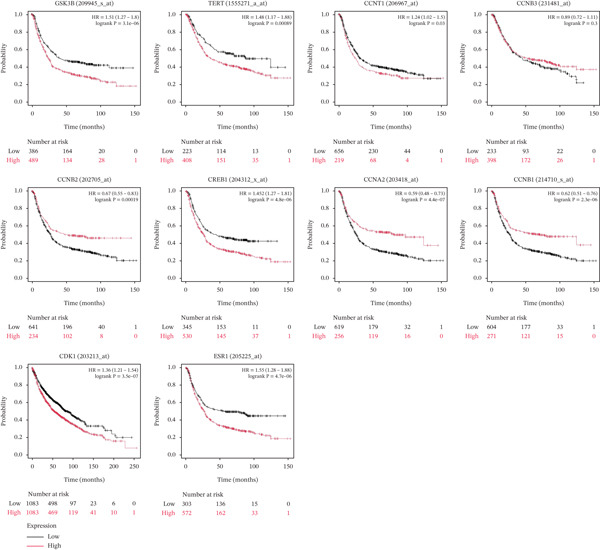
Kaplan–Meier survival analysis of hub genes in GC. Survival curves showing the association between hub gene expression and overall survival in GC patients.

Survival analysis based on the Kaplan–Meier Plotter website showed that high expression of GSK3B, TERT, CCNT1, CREB1, CDK1, and ESR1, as well as low expression of CCNB2, CCNA2, and CCNB1, was significantly associated with poor overall survival in GC patients (Figure 7). These results suggest that the expression levels of these genes may serve as potential prognostic biomarkers for GC.

### 3.6. Molecular Docking Results of ISO With Core Targets

**Figure 8:**
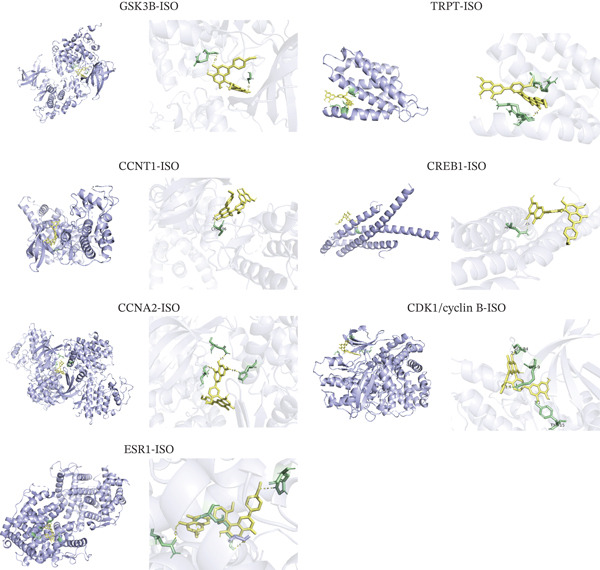
Molecular docking of ISO with core hub targets. Representative docking conformations of ISO with hub target proteins.

ISO was selected as the ligand, whereas 10 hub targets were chosen as receptors. Since CCNB3, CCNB2, CCNB1, and CDK1 belong to the CDK1/cyclin B complex, the ligand for all four targets is represented by the CDK1/cyclin B complex. It is generally accepted that a lower binding energy corresponds to a stronger binding likelihood between a ligand and its receptor. In the present study, all ligand–target binding energies were negative. ISO exhibited the strongest binding affinity toward CDK1/cyclin B, with a binding energy of −10.5 kcal/mol, whereas the weakest interaction was observed with CREB1, showing a binding energy of −8.1 kcal/mol. The binding energies between ISO and the other core targets were all less than −8 kcal/mol, suggesting stable and favorable docking interactions between ISO and the hub target proteins. Visualization of the docking conformations demonstrated the presence of several critical hydrogen bond interactions between ISO and the corresponding receptors, which contribute substantially to the strong binding affinity during the docking process, as illustrated in Figure 8. The detailed docking affinity results are listed in Table 2. In addition, MD simulations were conducted for two complexes with binding energies lower than −10 kcal/mol, namely CDK1/cyclin B–ISO and GSK3B–ISO. The results indicated that, after an initial conformational adjustment phase, both systems maintained stable binding modes throughout the simulation. Analyses of RMSD, Rg, and FEL consistently supported the presence of a dominant conformational cluster during the production phase. Furthermore, one to three hydrogen bonds between the protein and ligand were maintained during most of the simulation time, suggesting that ISO can stably bind within the corresponding binding pockets (Figures S1A, B, C, D, E, F and S2A, B, C, D, E, F).

**Table 2:** Binding energy between the receptor and the ligand.

Ligand	Receptor	Binding energy (kcal/mol)
Isoginkgetin	GSK3B	−10.1
Isoginkgetin	TERT	−8.5
Isoginkgetin	CCNT1	−9.7
Isoginkgetin	CREB1	−8.1
Isoginkgetin	CCNA2	−9.7
Isoginkgetin	CDK1/cyclin B	−10.5
Isoginkgetin	ESR1	−9.4

## 4. Discussion

GC remains a major global health challenge [1], and the discovery of novel therapeutic agents is crucial for improving patient outcomes. ISO, a natural flavonoid, has been reported to possess various pharmacological properties, including anticancer activity [6, 7], yet its precise molecular mechanisms in GC were not fully understood. In this study, we combined network pharmacology, bioinformatics, and molecular docking to systematically investigate the potential targets and mechanisms of ISO in GC.

Our analysis identified 66 overlapping genes between predicted ISO targets and differentially expressed genes in GC. Functional enrichment analyses revealed that these genes were mainly involved in cell cycle regulation, cellular senescence, reactive oxygen species metabolism, and the PI3K–Akt signaling pathway, suggesting that ISO may exert its antitumor effects by modulating multiple critical pathways associated with tumor growth and survival. Notably, the hub genes identified, including GSK3B, TERT, CCNT1, CCNB3, CCNB2, CREB1, CCNA2, CCNB1, CDK1, and ESR1. GSK3B: As a key regulator in cellular signaling, GSK3B participates in pathways such as Wnt/*β*‐catenin, regulating cell proliferation and apoptosis [19]. Aberrant activation of GSK3B in various cancers can promote tumor cell growth and drug resistance [20]. TERT: Responsible for telomere elongation, TERT maintains the unlimited proliferative capacity of cells [21]. The high activation of TERT is commonly observed in multiple malignancies and serves as a critical driver of tumor growth and recurrence [22]. CCNT1: Involved in transcriptional regulation and cell cycle progression, CCNT1 plays a role in cancer cell proliferation and apoptosis, particularly in regulating cell cycle progression [23–25]. CCNB1, CCNB2, and CCNB3 (cyclin B family members): These cyclins regulate the G2/M phase transition and are essential drivers of mitotic entry. Their overexpression is closely associated with rapid tumor cell proliferation [26–29]. CREB1: As a transcription factor, CREB1 regulates multiple genes related to proliferation and survival. It is highly expressed in various cancers and can promote cell proliferation, antiapoptotic activity, and invasiveness [30, 31]. CCNA2: Regulates the S phase and G2/M phase progression of the cell cycle. Its overexpression is often associated with tumor growth and apoptosis [32, 33]. CDK1: A key cell cycle regulatory kinase, CDK1 forms complexes with cyclin B to control the G2/M phase transition. Its high activity is closely linked to tumor proliferation [34–36]. ESR1: In addition to its classical role in breast cancer, ESR1 regulates metastasis and chemosensitivity in colorectal tumors [37, 38]. Its aberrant expression may influence the tumor microenvironment [39, 40]. Most of these genes were highly expressed in tumor tissues and showed positive correlations with each other, suggesting a coordinated role in promoting GC progression.

Survival analysis demonstrated that altered expression of several hub genes was significantly associated with poor overall survival, highlighting their potential prognostic value. Molecular docking results further confirmed stable interactions between ISO and these core targets, with the strongest binding observed with CREB1, suggesting a direct regulatory effect of ISO on key signaling proteins. These findings collectively support the notion that ISO may inhibit GC progression by targeting multiple hub genes and regulating cell cycle–related pathways.

While this study provides a comprehensive overview of the potential mechanisms of ISO in GC, further experimental validation is necessary. Functional studies, including in vitro and in vivo assays, are needed to confirm the regulatory effects of ISO on identified hub genes and signaling pathways. Additionally, further investigations into the pharmacokinetics, bioavailability, and safety profile of ISO are necessary to evaluate its potential for future translational research.

In conclusion, this study systematically explored the molecular targets and potential mechanisms of ISO in GC, providing a theoretical basis for further investigation. ISO′s multitargeted action on key cell cycle and signaling pathways indicates its potential relevance in GC.

## Author Contributions

L.L. collected data, analyzed relevant data, and drafted the manuscript; H.Z. collected data and revised the manuscript; K.C. collected data and validated results. H.C. revised and edited the manuscript. L.L., H.Z., and K.C contributed equally to this work and should be regarded as cofirst authors.

## Funding

No funding was received for this manuscript.

## Disclosure

All authors read and approved the final manuscript. They warrant that the article is the authors′ original work, has not received prior publication, and is not under consideration for publication elsewhere.

## Ethics Statement

The authors have nothing to report.

## Conflicts of Interest

The authors declare no conflicts of interest.

## Supporting Information

Additional supporting information can be found online in the Supporting Information section.

## Supporting information


**Supporting Information 1** Figure S1: Comprehensive analysis of the 100 ns molecular dynamics trajectory of the CDK1/cyclin B–ISO complex. (A) RMSD; (B) RMSF; (B) radius of gyration (Rg); (D) protein–ligand hydrogen bond number; (E) solvent‐accessible surface area (SASA); and (F) free energy landscape constructed based on RMSD and Rg.

**Supporting Information 2** Figure S2: Comprehensive analysis of the 100 ns molecular dynamics trajectory of the GSK3B–ISO complex. (A) RMSD; (B) RMSF; (C) radius of gyration (Rg); (D) protein–ligand hydrogen bond number; (E) solvent‐accessible surface area (SASA); and (F) free energy landscape constructed based on RMSD and Rg.

**Supporting Information 3** Table S1: Target genes identified by SwissTargetPrediction and the Similarity Ensemble Approach (SEA).

**Supporting Information 4** Table S2: Common genes between ISO target genes and gastric cancer–related genes.

## Data Availability

The datasets supporting the conclusions of this article are available in the public databases TCGA database (https://portal.gdc.cancer.gov/) project TCGA‐STAD, SwissTargetPrediction (http://www.swisstargetprediction.ch/), and the Similarity Ensemble Approach (SEA, http://sea.bkslab.org/).
