# Supplementary figures and images for: Network Pharmacology‐Based Identification of Potential Targets and Mechanisms of Isoginkgetin in Gastric Cancer
Source: Biomed Res Int. 2026 Jun 16;2026:8863892. doi: 10.1155/bmri/8863892 (PMC13271043; doi:10.1155/bmri/8863892)

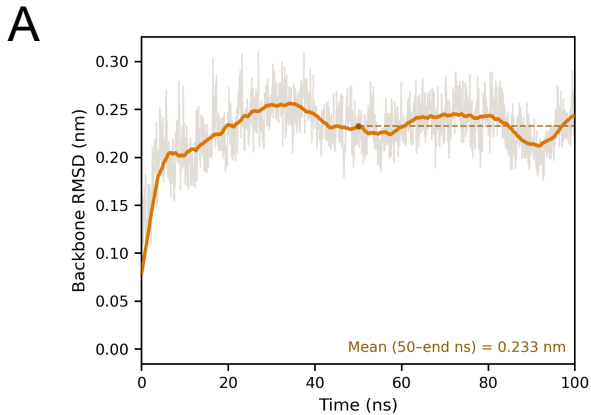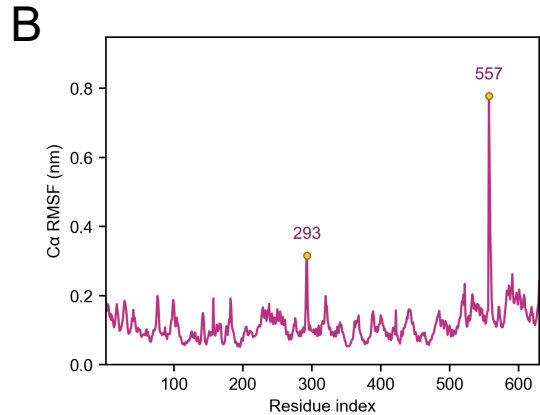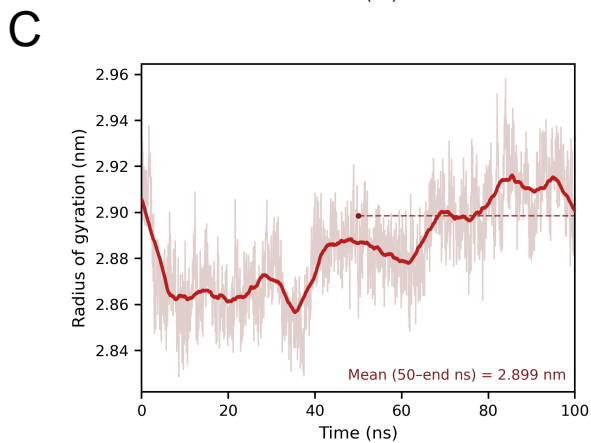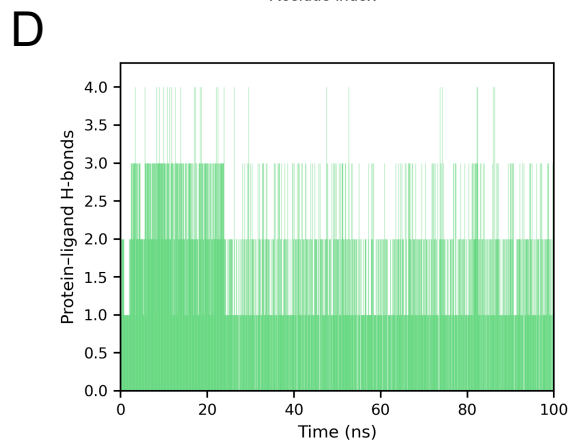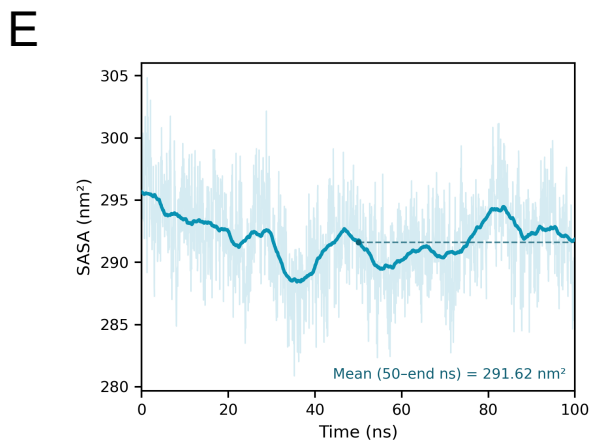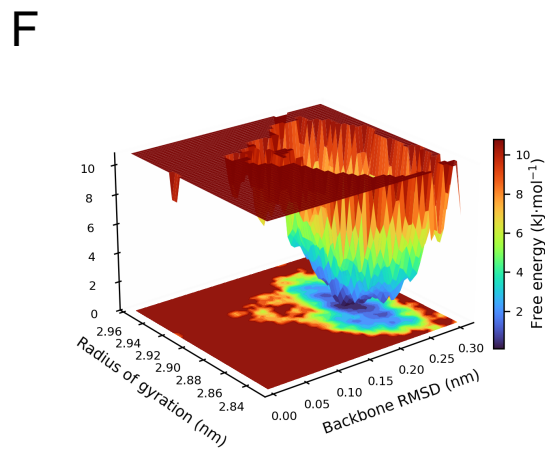

Supplement: Supplementary file 1 — Supporting Information 1 Figure S1: Comprehensive analysis of the 100 ns molecular dynamics trajectory of the CDK1/cyclin B–ISO complex. (A) RMSD; (B) RMSF; (B) radius of gyration (Rg); (D) protein–ligand hydrogen bond number; (E) solvent‐accessible surface area (SASA); and (F) free energy landscape constructed based on RMSD and Rg. [file BMRI-2026-8863892-s001.pdf]

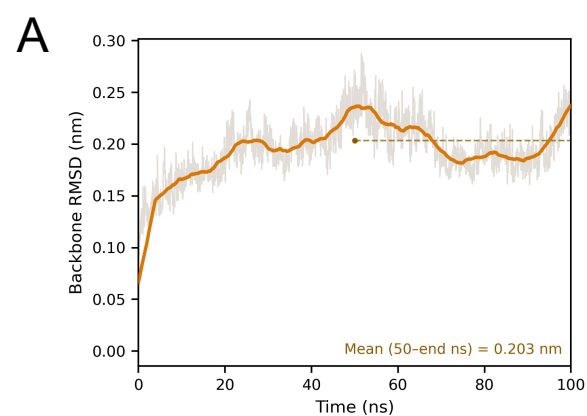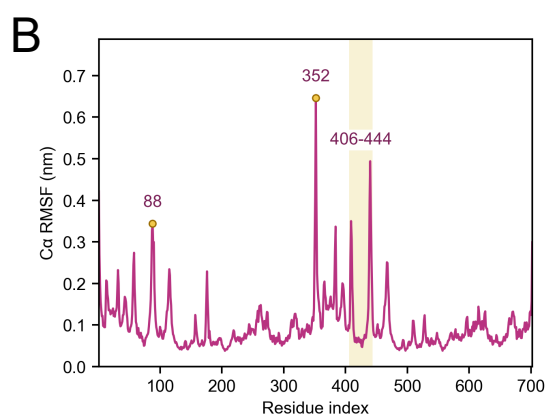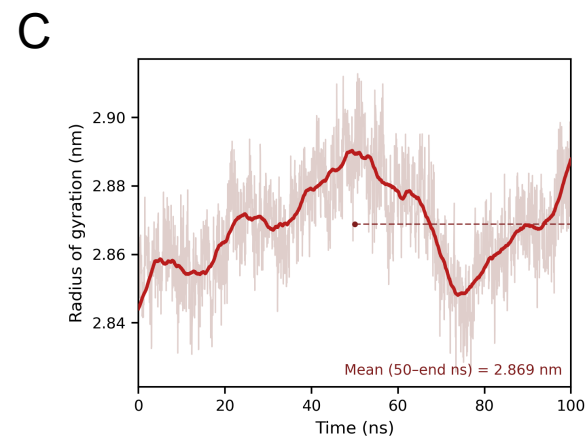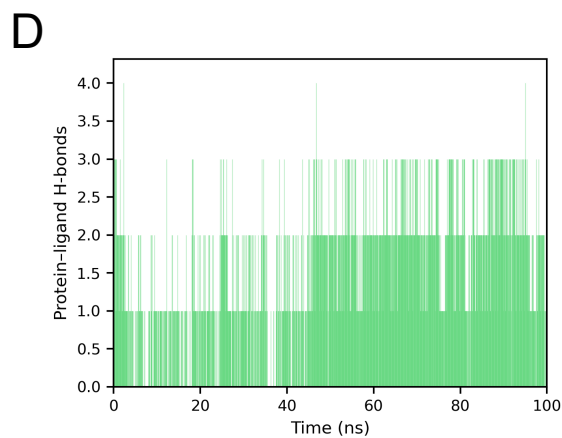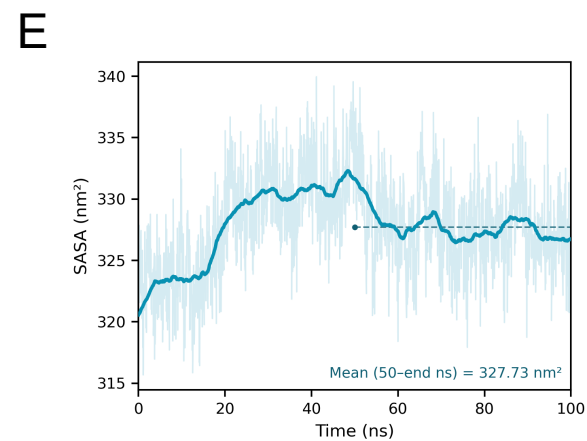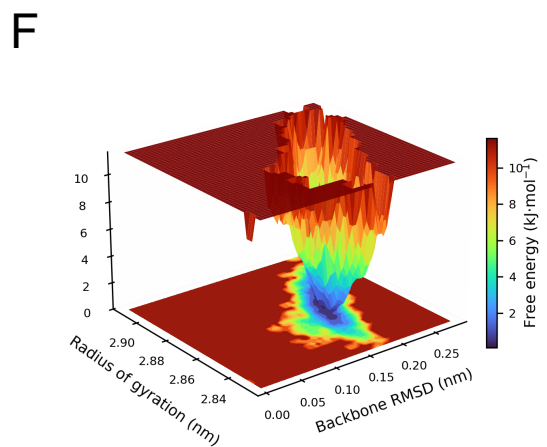

Supplement: Supplementary file 2 — Supporting Information 2 Figure S2: Comprehensive analysis of the 100 ns molecular dynamics trajectory of the GSK3B–ISO complex. (A) RMSD; (B) RMSF; (C) radius of gyration (Rg); (D) protein–ligand hydrogen bond number; (E) solvent‐accessible surface area (SASA); and (F) free energy landscape constructed based on RMSD and Rg. [file BMRI-2026-8863892-s002.pdf]
