# Supplementary material for: Network Pharmacology‐Based Identification of Potential Targets and Mechanisms of Isoginkgetin in Gastric Cancer
Source: Biomed Res Int. 2026 Jun 16;2026:8863892. doi: 10.1155/bmri/8863892 (PMC13271043; doi:10.1155/bmri/8863892)
Supplement: Supplementary file 3 — Supporting Information 3 Table S1: Target genes identified by SwissTargetPrediction and the Similarity Ensemble Approach (SEA). [file BMRI-2026-8863892-s003.docx]

Supplementary Table S1. Target genes identified by SwissTargetPrediction and the Similarity Ensemble Approach (SEA)

| Gene name | Gene |
| --- | --- |
| Beta-secretase 1 | BACE1 |
| Transitional endoplasmic reticulum ATPase | VCP |
| Placenta growth factor | PGF |
| Vascular endothelial growth factor A | VEGFA |
| GABA-A receptor; alpha-1/beta-2/gamma-2 | GABRA1 GABRB2 GABRG2 |
| Serotonin 2c (5-HT2c) receptor | HTR2C |
| Dopamine D3 receptor | DRD3 |
| Delta opioid receptor | OPRD1 |
| Protein-tyrosine phosphatase 1B | PTPN1 |
| Cyclin-dependent kinase 5/CDK5 activator 1 | CDK5R1 CDK5 |
| Induced myeloid leukemia cell differentiation protein Mcl-1 | MCL1 |
| Cytochrome P450 1B1 | CYP1B1 |
| NEDD8-activating enzyme E1 regulatory subunit | NAE1 |
| Bcl-2-related protein A1 | BCL2A1 |
| Tankyrase-2 | TNKS2 |
| Tankyrase-1 | TNKS |
| Stem cell growth factor receptor | KIT |
| Adenosine A1 receptor (by homology) | ADORA1 |
| Adenosine A3 receptor | ADORA3 |
| Serine/threonine-protein kinase PIM1 | PIM1 |
| Adenosine A2a receptor (by homology) | ADORA2A |
| NADPH oxidase 4 | NOX4 |
| Aldose reductase (by homology) | AKR1B1 |
| Monoamine oxidase A | MAOA |
| Tyrosine-protein kinase receptor FLT3 | FLT3 |
| Cytochrome P450 19A1 | CYP19A1 |
| Cyclin-dependent kinase 1/cyclin B | CCNB3 CDK1 CCNB1 CCNB2 |
| Cyclooxygenase-2 | PTGS2 |
| Estrogen receptor beta | ESR2 |
| Cyclin-dependent kinase 6 | CDK6 |
| Tyrosine-protein kinase SYK | SYK |
| Glycogen synthase kinase-3 beta | GSK3B |
| Transthyretin | TTR |
| Casein kinase II alpha | CSNK2A1 |
| Cystic fibrosis transmembrane conductance regulator | CFTR |
| Aldo-keto reductase family 1 member B10 | AKR1B10 |
| Tyrosinase | TYR |
| Arachidonate 5-lipoxygenase | ALOX5 |
| Aryl hydrocarbon receptor | AHR |
| Estrogen-related receptor alpha | ESRRA |
| Xanthine dehydrogenase | XDH |
| Receptor-type tyrosine-protein phosphatase S | PTPRS |
| AMY1C | AMY1A |
| G-protein coupled receptor 35 | GPR35 |
| Death-associated protein kinase 1 | DAPK1 |
| DNA-3-methyladenine glycosylase | MPG |
| Solute carrier family 22 member 12 | SLC22A12 |
| Carbonyl reductase [NADPH] 1 | CBR1 |
| Cyclic AMP-responsive element-binding protein 1 | CREB1 |
| Cystathionine beta-synthase | CBS |
| Anthrax toxin receptor 2 | ANTXR2 |
| ELAV-like protein 3 | ELAVL3 |
| Xanthine dehydrogenase/oxidase | XDH |
| Potassium voltage-gated channel subfamily D member 3 | KCND3 |
| Broad substrate specificity ATP-binding cassette transporter ABCG2 | ABCG2 |
| Alpha-amylase 1 | AMY1A |
| ATP-dependent translocase ABCB1 | ABCB1 |
| Multidrug resistance-associated protein 1 | ABCC1 |
| Cytochrome P450 1A1 | CYP1A1 |
| Taste receptor type 2 member 31 | TAS2R31 |
| [Pyruvate dehydrogenase (acetyl-transferring)] kinase isozyme 4, mitochondrial | PDK4 |
| Serine/threonine-protein kinase VRK2 | VRK2 |
| Interleukin-2 | IL2 |
| Lactoylglutathione lyase | GLO1 |
| CDK-activating kinase assembly factor MAT1 | MNAT1 |
| ELAV-like protein 1 | ELAVL1 |
| [Pyruvate dehydrogenase (acetyl-transferring)] kinase isozyme 3, mitochondrial | PDK3 |
| Poly [ADP-ribose] polymerase tankyrase-1 | TNKS |
| Telomerase reverse transcriptase | TERT |
| Ornithine decarboxylase | ODC1 |
| Monocarboxylate transporter 4 | SLC16A3 |
| Serine/threonine-protein kinase MAK | MAK |
| G protein-coupled receptor kinase 6 | GRK6 |
| Activin receptor type-2A | ACVR2A |
| Calmodulin-1 | CALM1 |
| Amyloid-beta precursor protein | APP |
| Estrogen receptor | ESR1 |
| Glutathione S-transferase omega-1 | GSTO1 |
| Serine/threonine-protein kinase N1 | PKN1 |
| Cyclin-dependent kinase-like 5 | CDKL5 |
| Calcium/calmodulin-dependent protein kinase type IV | CAMK4 |
| Cytochrome P450 2C8 | CYP2C8 |
| Lysine-specific demethylase 4E | KDM4E |
| Carbonic anhydrase 7 | CA7 |
| Cyclin-dependent kinase 13 | CDK13 |
| Casein kinase I isoform alpha-like | CSNK1A1L |
| Serine/threonine-protein kinase MRCK beta | CDC42BPB |
| Serine/threonine-protein kinase 32A | STK32A |
| Carbonic anhydrase 4 | CA4 |
| Cyclin-dependent kinase 5 activator 1 | CDK5R1 |
| Phosphatidylinositol 4-phosphate 5-kinase type-1 gamma | PIP5K1C |
| Poly [ADP-ribose] polymerase tankyrase-2 | TNKS2 |
| Malate dehydrogenase, cytoplasmic | MDH1 |
| Aldo-keto reductase family 1 member B1 | AKR1B1 |
| Serine/threonine-protein kinase Nek6 | NEK6 |
| Protein disulfide-isomerase | P4HB |
| Beta-galactoside alpha-2,6-sialyltransferase 1 | ST6GAL1 |
| Amine oxidase [flavin-containing] A | MAOA |
| Cyclin-dependent kinase 15 | CDK15 |
| Cyclin-T1 | CCNT1 |
| Endoplasmic reticulum aminopeptidase 1 | ERAP1 |
| Acetylcholinesterase | ACHE |
| Activin receptor type-2B | ACVR2B |
| Cyclin-dependent kinase 18 | CDK18 |
| Cyclin-A2 | CCNA2 |
| Arachidonate 12-lipoxygenase, 12S-type | ALOX12 |
| Serine/threonine-protein kinase ICK | CILK1 |
