# Supplementary material for: Network Pharmacology‐Based Identification of Potential Targets and Mechanisms of Isoginkgetin in Gastric Cancer
Source: Biomed Res Int. 2026 Jun 16;2026:8863892. doi: 10.1155/bmri/8863892 (PMC13271043; doi:10.1155/bmri/8863892)
Supplement: Supplementary file 4 — Supporting Information 4 Table S2: Common genes between ISO target genes and gastric cancer–related genes. [file BMRI-2026-8863892-s004.docx]

Supplementary Table S2. common genes between ISO target genes and gastric cancer–related genes

| Gene name | Gene |
| --- | --- |
| Beta-secretase 1 | BACE1 |
| Placenta growth factor | PGF |
| Vascular endothelial growth factor A | VEGFA |
| GABA-A receptor; alpha-1/beta-2/gamma-2 | GABRA1 |
| Serotonin 2c (5-HT2c) receptor | HTR2C |
| Delta opioid receptor | OPRD1 |
| Cyclin-dependent kinase 5 activator 1 | CDK5R1 |
| Induced myeloid leukemia cell differentiation protein Mcl-1 | MCL1 |
| Cytochrome P450 1B1 | CYP1B1 |
| NEDD8-activating enzyme E1 regulatory subunit | NAE1 |
| Bcl-2-related protein A1 | BCL2A1 |
| Tankyrase-1 | TNKS |
| Stem cell growth factor receptor | KIT |
| Adenosine A1 receptor (by homology) | ADORA1 |
| Serine/threonine-protein kinase PIM1 | PIM1 |
| NADPH oxidase 4 | NOX4 |
| Monoamine oxidase A | MAOA |
| Cytochrome P450 19A1 | CYP19A1 |
| Cyclin-dependent kinase 1/cyclin B | CCNB3 |
| Cyclin-dependent kinase 6 | CDK6 |
| Glycogen synthase kinase-3 beta | GSK3B |
| Casein kinase II alpha | CSNK2A1 |
| Cystic fibrosis transmembrane conductance regulator | CFTR |
| Aldo-keto reductase family 1 member B10 | AKR1B10 |
| Aryl hydrocarbon receptor | AHR |
| Estrogen-related receptor alpha | ESRRA |
| Xanthine dehydrogenase | XDH |
| Receptor-type tyrosine-protein phosphatase S | PTPRS |
| DNA-3-methyladenine glycosylase | MPG |
| Solute carrier family 22 member 12 | SLC22A12 |
| Carbonyl reductase [NADPH] 1 | CBR1 |
| Cyclin-dependent kinase 1/cyclin B | CDK1 |
| Cyclin-dependent kinase 1/cyclin B | CCNB1 |
| Cyclin-dependent kinase 1/cyclin B | CCNB2 |
| Cyclic AMP-responsive element-binding protein 1 | CREB1 |
| Anthrax toxin receptor 2 | ANTXR2 |
| ELAV-like protein 3 | ELAVL3 |
| Potassium voltage-gated channel subfamily D member 3 | KCND3 |
| Broad substrate specificity ATP-binding cassette transporter ABCG2 | ABCG2 |
| ATP-dependent translocase ABCB1 | ABCB1 |
| Cytochrome P450 1A1 | CYP1A1 |
| [Pyruvate dehydrogenase (acetyl-transferring)] kinase isozyme 4, mitochondrial | PDK4 |
| Serine/threonine-protein kinase VRK2 | VRK2 |
| Interleukin-2 | IL2 |
| ELAV-like protein 1 | ELAVL1 |
| [Pyruvate dehydrogenase (acetyl-transferring)] kinase isozyme 3, mitochondrial | PDK3 |
| Telomerase reverse transcriptase | TERT |
| Ornithine decarboxylase | ODC1 |
| Monocarboxylate transporter 4 | SLC16A3 |
| Serine/threonine-protein kinase MAK | MAK |
| Activin receptor type-2A | ACVR2A |
| Calmodulin-1 | CALM1 |
| Estrogen receptor | ESR1 |
| Glutathione S-transferase omega-1 | GSTO1 |
| Carbonic anhydrase 7 | CA7 |
| Cyclin-dependent kinase 13 | CDK13 |
| Serine/threonine-protein kinase 32A | STK32A |
| Phosphatidylinositol 4-phosphate 5-kinase type-1 gamma | PIP5K1C |
| Malate dehydrogenase, cytoplasmic | MDH1 |
| Serine/threonine-protein kinase Nek6 | NEK6 |
| Protein disulfide-isomerase | P4HB |
| Cyclin-dependent kinase 15 | CDK15 |
| Cyclin-T1 | CCNT1 |
| Activin receptor type-2B | ACVR2B |
| Cyclin-A2 | CCNA2 |
| Arachidonate 12-lipoxygenase, 12S-type | ALOX12 |
